# Effects of mask-wearing on the inhalability and deposition of airborne SARS-CoV-2 aerosols in human upper airway

**DOI:** 10.1063/5.0034580

**Published:** 2020-12-01

**Authors:** Jinxiang Xi, Xiuhua April Si, Ramaswamy Nagarajan

**Affiliations:** 1 Department of Biomedical Engineering, University of Massachusetts at Lowell, 1 University Ave., Lowell, Massachusetts 01854, USA; 2 Department of Aerospace, Industrial, and Mechanical Engineering, California Baptist University, 8432 Magnolia Ave., Riverside, California 92504, USA; 3 Department of Plastics Engineering, University of Massachusetts at Lowell, 1 University Ave., Lowell, Massachusetts 01854, USA; 4 Fabric Discovery Center, University of Massachusetts at Lowell, 110 Canal St., Lowell, Massachusetts 01852, USA

## Abstract

Even though face masks are well accepted as tools useful in reducing COVID-19 transmissions, their effectiveness in reducing viral loads in the respiratory tract is unclear. Wearing a mask will significantly alter the airflow and particle dynamics near the face, which can change the inhalability of ambient particles. The objective of this study is to investigate the effects of wearing a surgical mask on inspiratory airflow and dosimetry of airborne, virus-laden aerosols on the face and in the respiratory tract. A computational model was developed that comprised a pleated surgical mask, a face model, and an image-based upper airway geometry. The viral load in the nose was particularly examined with and without a mask. Results show that when breathing without a mask, air enters the mouth and nose through specific paths. When wearing a mask, however, air enters the mouth and nose through the entire surface of the mask at lower speeds, which favors the inhalation of ambient aerosols into the nose. With a 65% filtration efficiency (FE) typical for a three-layer surgical mask, wearing a mask reduces dosimetry for all micrometer particles except those of size 1 *µ*m–3 *µ*m, for which equivalent dosimetry with and without a mask in the upper airway was predicted. Wearing a mask reduces particle penetration into the lungs, regardless of the FE of the mask. The results also show that mask-wearing protects the upper airway (particularly the nose and larynx) best from particles larger than 10 *µ*m while protecting the lungs best from particles smaller than 10 *µ*m.

## I. INTRODUCTION

Infectious respiratory diseases spread when a healthy person comes in contact with virus-laden droplets from someone who has been infected, often through a sneeze or cough.^1,2^ Wearing a mask has been proven to be an effective method of protection in this pandemic, which both reduces the exhalation of virus-laden aerosols from a COVID patient and minimizes the inhalation of airborne virus-laden aerosols by the subjects surrounding the patient.^3,4^ Masks are available with different filtration efficiencies and levels of breathability. The filtration media are often made of micrometer or nano-sized fibers, arranged as a matrix or network, to achieve the desired filtration efficiency (FE).^5^ A mask with a higher FE often has a higher breathing resistance, i.e., worse breathability.

Physiological studies show that the SARS-CoV-2 virus that causes COVID-19 first deposits in the human upper airway to cause infection of the nasal goblet secretory cells and then spreads to the central and inner parts of the lungs.^6,7^ The final target is the alveoli, the smallest respiration units that has a diameter of 0.2 mm–0.4 mm.^8,9^ The virus will attack the type-II cells in the alveoli and interfere with their capacity to secrete the surfactants needed to maintain normal breathing.^6^ With an inadequate lining of surfactants on the alveolar wall, water surface tension can increase the breathing effort by two to four times to draw in the same volume of fresh air (or oxygen).^10^ To make things worse, the coincidence of hypertension of the cardiovascular system can fill the alveolar airspace with fluids, making breathing and oxygen exchange even harder.^11^ Usually under this condition, mechanical ventilation to the patients is needed.

There exists a threshold number of SARS-CoV-2 viruses (i.e., the infectious dose in both concentration and time) that are necessary to cause the illness.^12,13^ It is currently unclear what the exact infectious dose is for COVID-19, but it is estimated to be 1000 viruses, by analogy to influenza and SARS.^14–17^ Therefore, the knowledge of the local deposition rates of the virus-laden particles on the epithelial cells (i.e., viral loads) is crucial in determining the risk of COVID infection. Due to face covering, both inhaled and exhaled airflow can be altered significantly. Simha and Rao visualized the expiratory flows of coughs and quantified the propagation distances with and without a mask using a Schlieren imaging system. It was demonstrated that the cough flow fields were governed by the propagation of viscous vortex rings. Verma *et al.*^19,20^ used a laser-illuminating system to visualize the effectiveness of face masks and face shields in obstructing respiratory jets. Their results confirmed that a well-fitted mask can significantly curtail the speed and range of expelled droplets while a face shield still allowed droplets to move around and spread out over a large area.^19,20^ However, how the presence of a mask affects the inhalation dosimetry of ambient aerosols in the upper airway is not clear, even though we expect certain degrees of difference from that without wearing a mask. In contrast to the recent resurgence of interest in using Schlieren and (particle image velocimetry) systems to visualize expiratory flows,^18–22^ reports of inspiratory flow and particle dynamics with face-covering are scarce, with the exception of a very recent study by Dbouk and Drikakis,^23^ who elegantly simulated the effectiveness of face-covering on reducing airborne viral infections. This lack of investigation into inhalation dosimetry with masks can be largely attributed to the inaccessibility of measurement within the mask and in the human respiration tract. However, considering the large variations in inspiratory airflows caused by wearing a mask, it is hypothesized that the inhalability of airborne particles into the nose/mouth, as well as their subsequent deposition within the upper respiratory tract and lungs, can also be significantly different.

The objective of this study is to numerically characterize the difference in the deposition distribution of ambient aerosols in the upper airway with and without a mask. The specific aims include:

(1) developing a computational model that includes a mask, face, and upper airway with a perfect mask–face seal,
(2) studying the inspiratory airflows and particle motions near the face when wearing a surgical mask in comparison to those without a mask,
(3) characterizing the effect of particle size, inhalation flow rate, and mask resistance matrix on the dosimetry of ambient aerosols with and without a mask, and
(4) quantifying the fraction of airborne particles deposited on the face, retained in the upper airway, and entering the lungs; regional deposition in the nose and larynx will be particularly examined.

The results of this study will provide insights into the airflows and particle dynamics with a mask on and the factors involved in determining the protection efficacy of face-covering, which is an area remaining largely unexplored but will be of high interest to patients, care providers, PPE designers, and public policymakers.

## II. METHODS

### A. Mask–face-airway model

**FIG. 1.**
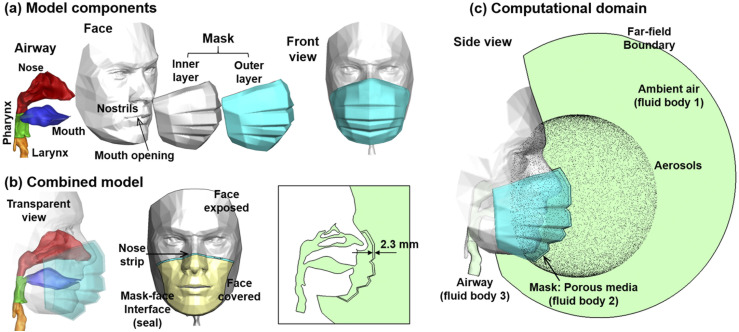
Mask–face-airway model: (a) model components: upper airway (nose, mouth, pharynx, and larynx), face with nostrils and mouth opening, and mask with inner and out layers; (b) the combined model with the upper airway being connected to the face and the mask fitted on the face with no leakage; and (c) the computational domain with spherical ambient airspace and a spherical aerosol profile. There are three fluid bodies: ambient air (fluid body 1), mask (fluid body 2 as porous media), and airway (fluid body 3). The face was divided into (1) face exposed and (2) face covered [yellow color in (b)], delineated by the mask–face interface (seal) and the nasal strip.

The computational model consisted of three parts: a realistic model of a disposable three-layer surgical mask, a face model with spherical ambient airspace, and an upper airway model with the nose, mouth, pharynx, and larynx [Fig. 1(a)]. The upper airway model was connected to the face seamlessly by fusing the nostrils and mouth opening to the corresponding regions of the face [Fig. 1(b)]. The mask model was then appended to the face by covering the mouth and nose and was fit tightly to the face. The mask–face interface is shown in Fig. 1(b) as a close-loop strip (light blue), within which the face will be covered by the mask [yellow color, Fig. 1(b)], and the rest of the face is exposed to the environment.

Individually, the upper airway geometry was a combination of a nasal cavity, an oral cavity, and a pharyngolaryngeal region, which were developed separately in our previous studies. In brief, the nose model was reconstructed from MRI scans of a 53-year-old subject with no rhinology disorders.^24,25^ The oral cavity was modified from the mouth model developed by Xi and Longest,^26^ which was further based on an oral cast reconstructed from a dental impression by Cheng *et al.*^27^ The surgical mask model was specifically developed for this study using open-source 3D rendering software Blender (Blender Foundation, Amsterdam, the Netherlands) based on photos of a subject wearing a disposable three-layer surgical mask from different angles. Morphological details, such as the three folds (or pleats) on the mask, were retained. To avoid the compounding effect of mask–face sealing effects, a perfect mask–face fit was assumed in this study. In doing so, the mask model as a volume was extended backward to intersect with the human face. By deleting the parts of the mask that fall behind the face, a seamless mask–face seal was achieved [Fig. 1(b)].

### B. Flow-particle modeling

There are three fluid bodies: ambient air (fluid body 1), mask (fluid body 2), and airway (fluid body 3), as shown in Fig. 1(c). Porous media were adopted to simulate the resistance of the mask, whose properties are described in detail in Sec. II C. Airflow was assumed to be incompressible (ρ = 1.204 kg/m^3^) and isothermal (20 °C) with a dynamic viscosity of 1.825 × 10^−5^ kg/m s. To simulate inspiratory airflows, zero gauge pressure was specified at the far-field boundary, and a negative flow rate was specified at the trachea opening [Fig. 1(c)]. The airflow was drawn in by the negative pressures in the airway from the ambient airspace, through the mask, and into the oronasal openings, with a pressure drop across the mask and modified airflows around the mask. Considering the multiple flow regimes that may exist in the ambient airspace and respiratory tract, the low Reynolds number (LRN) *k*-*ω* turbulence model was used to simulate the inspiratory airflows based on its ability to accurately predict pressure, velocity, and shear for transitional and turbulent flows.^28^ Moreover, it has been demonstrated to provide an accurate solution for laminar flow as the turbulent viscosity approaches zero.^29^ For more details of the LRN *k*-*ω* turbulence model, refer to the work of Wilcox.^29^

To simulate the inhalation dosimetry of airborne aerosols, a spherical profile of monodisperse particles was generated that surrounded the face with an approximate distance of 0.10 m. The particle motions were tracked using a discrete phase Lagrangian tracking algorithm enhanced with user-defined functions (UDFs) for the near-wall treatment of flow and particle velocities.^30,31^ Physical properties of the particles include a spherical shape and a particle density *ρ*_*p*_ of 1.0 g/cm^3^. The transport equations are expressed below:

$$\frac{dv_{i}}{dt}=\alpha \frac{Du_{i}}{Dt}+\frac{f}{\tau_{p}}\left(u_{i}-v_{i}\right)+g_{i}\left(1-\alpha \right);\;and\;\frac{dx_{i}}{dt}=v_{i}\left(t\right),$$ (1)

Here, *v*_*i*_ and *u*_*i*_ are particle and flow velocities, respectively, *α* is the flow-particle density ratio, *f* is the drag factor, and *τ*_*p*_ = *ρ*_*p*_*d*_*p*_^2^/18μ is the particle relaxation time (i.e., the time for a particle to respond to local flow changes). One-way coupling (from flow to the particle) was assumed for ambient aerosols ranging from 1 *μ*m to 20 *µ*m with main flow tracking. This UDF-enhanced Lagrangian particle-tracking model had shown high fidelity in matching *in vitro* dosimetry of both nanometer^32,33^ and micrometer particles.^34,35^

### C. Mask material properties

A mask is characterized by its filtration efficiency (FE) and permeability (or breathability). It is noted that even though a high-filtration mask often has a high flow resistance, these two parameters can be independent of each other. A surgical mask with an experimentally measured FE (65%) and porosity (10%) was used in this study,^36^ where the FE was measured using TSI 8130 (TSI Incorporated) and the porosity was quantified using SEM images of the mask sample. A mask FE of 0%, where all particles passed through the mask with no deposition, was also considered to represent the worst scenario of wearing a mask. Considering a 0% FE mask also allows the study of the impact of the mask-altered flow field alone on particle inhalability. It is noted that particle deposition in the mask cannot be directly simulated in ANSYS Fluent (Canonsburg, PA); rather, all particles that come into contact with the mask will pass through it. Post-processing was needed to calculate the deposition fraction (DF) on the face separately, as well as in the upper airway and lungs. For a 65% FE mask, the facial DF was calculated by adding two groups of particles: all particles depositing on the uncovered face [gray, Fig. 1(b)] and 35% of the particles depositing on the mask-covered face [yellow, Fig. 1(b)]. For the DFs in the upper airway and lungs, only 35% of the deposited particles were counted since 65% were filtered out by the mask.

The viscous resistance of the mask in the normal direction was calculated from Darcy’s law using the flow rate (85 l/min) and pressure (∼96 Pa) measured by TSI 8130^36,37^ as follows:

$$Viscous\text{ Resistance}=\frac{\Delta P}{\left(Q/A\right)\mu L}.$$ (2)

The viscous resistance was calculated as a bulk value of 8.864 × 10^9^ 1/m^2^ based on a sampling area *A* = 55 cm^2^, a dynamic viscosity *μ* = 1.825 × 10^−5^ kg/m s, and an overall mask thickness *L* = 2.3 mm without differentiating the three layers that comprise the surgical mask [Fig. 1(b)]. It is noted that the resistance of the mask can be different in lateral directions. To investigate the resistance matrix effects on airflow and particle dynamics near the face and inside the airways, five resistance matrices were considered, with the lateral viscous resistance many times that in the normal direction. These include 1-1-1, 3-1-3, 6-1-6, 10-1-10, and 10-10-10, with “1” representing the normal resistance (8.864 × 10^9^ 1/m^2^), “3” representing three times that of the normal resistance (2.659 × 10^10^ 1/m^2^), and so on. The mask resistance matrix 1-3-1 was used as the control case and was examined for all particle sizes (1 *µ*m–20 *µ*m) and four flow rates (15 l/min, 30 l/min, 45 l/min, and 60 l/min); however, for the other four resistance matrices, only 30 l/min was considered. For all simulations with a mask, a case without a mask was simulated to understand the effects of mask-wearing in different scenarios.

### D. Numerical methods

**FIG. 2.**
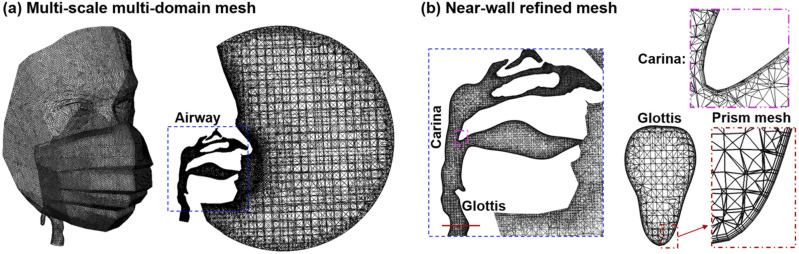
Computational mesh: (a) multi-scale, multi-domain mesh, with coarse mesh in the ambient airspace, fine mesh on the face and mask, and ultrafine mesh in the airway and (b) body-fitted mesh was used in the near-wall region of the airway, with four layers of prismatic cells and a height of 0.03 mm in the first layer, as displayed in the oropharyngeal carina and the glottis.

ANSYS Fluent (Canonsburg, PA) was used to simulate inspiratory airflows and particle motions. User-defined functions were implemented to specify the airborne aerosol distribution, calculate the deposition fractions (DFs), and plot the spatial distribution of deposited particles.^38^ The deposition enhancement factor (DEF), which represents the ratio of local DF over the average DF, was used to visualize the intensity of deposition at a cellular level.^39^ Considering the large size differences among the ambient airspace, mask, and airway, a multi-scale, multi-domain mesh was generated using ANSYS ICEM CFD (Ansys, Inc.) [Fig. 2(a)]. To capture the near-wall velocity variation, a four-layer body-fitted prismatic mesh was specified near the face and in the airway, with a height of 0.03 mm in the first layer [Fig. 2(b)]. A grid-independent study was conducted using six mesh densities, i.e., 1.13 × 10^6^, 1.87 × 10^6^, 2.98 × 10^6^, 3.97 × 10^6^, 4.93 × 10^6^, and 6.24 × 10^6^. The grid-independent results were achieved at 4.93 × 10^6^, where the variation in the nasal deposition fraction was less than 1% relative to that at 6.24 × 10^6^. Considering that quantifying the particle deposition fraction is inherently a statistical process, a sufficient number of sample particles are needed to attain statistically converged (i.e., particle-count independent) deposition results. In doing so, the number of released particles was increased from 10 000 to 100 000, with an increment of 5000 particles. The particle-count independent results were achieved at 60 000 particles when the variation in the nasal deposition was less than 0.5% between two consecutive tests.

## III. RESULTS

### A. Airflow and particle dynamics with and without a mask

#### 1. Airflow

**FIG. 3.**
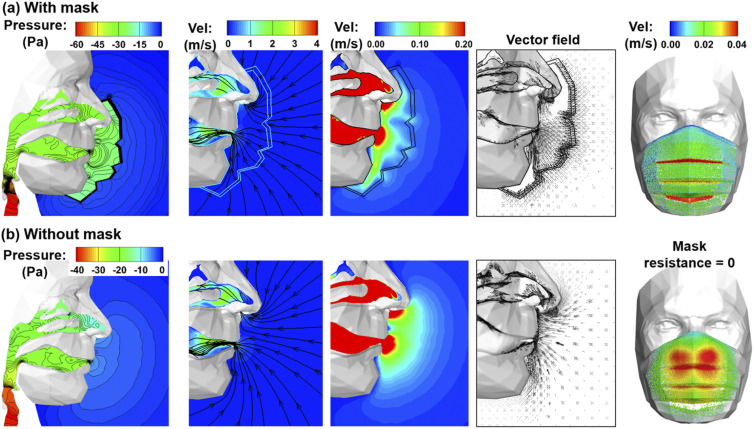
Comparison of inspiratory airflows at 30 l/min between two scenarios, (a) with a mask and (b) without a mask in terms of the mid-plane pressure contour (first panel), velocity contours and streamlines at large (second panel) and small (third panel) scales, vector field (fourth panel), and velocity of fluid particles passing the mask (fifth panel). Wearing a mask significantly distorted the airflow and pressure distributions.

Wearing a mask can notably distort the inhalation aerodynamics in comparison to that without a mask. Figure 3 shows the comparison between inspiratory airflow and pressure fields at 30 l/min with and without a mask in terms of pressure distributions, velocity contours, streamlines, and vector fields near the oronasal openings. As expected, wearing a mask caused an abrupt pressure drop of 22 Pa across the mask, which had a resistance matrix of 3*a*-*a*-3*a*, where *a* = 8.864 × 10^9^ 1/m^2^. As a result, the total pressure drop between the ambient and trachea was about 20 Pa higher with a mask than without it (leftmost panels, Fig. 3). In addition, flow streamlines across the mask were notably refracted (i.e., turning aside from their straight paths with various angles), especially in the vicinity of the mask pleats (or folds), as shown in the second panel of Fig. 3(a). By comparison, all streamlines entering the airway without a mask are smooth. These flow distortions from a mask were further illustrated by the velocity contour in the mid-plane [third panel of Fig. 3(a)]. With a range of 0.0 m/s–0.2 m/s, the airflow speeds are noticeably higher near the mask pleats than other regions (i.e., smooth surface) of the mask, which are clearly different from the smooth velocity contours without a mask. The fourth panel compares the vector fields at the mid-plane with and without a mask. One apparent difference was the higher flow speed near the mask pleats, indicating a significant impact from the physical properties of the mask (i.e., shape, size, resistance, orientation, etc.). As these mask folds are located below the mouth and nose, the flow entering the nose after a mask appears more aligned with the nostril orientation than that without a mask (fourth panel, Fig. 3).

The rightmost panels of Fig. 3(a) visualize the velocities of airflow through a mask with massless particles at an inhalation flow rate of 30 l/min. To simulate the scenario without a mask, the porous media resistances were specified as zero [rightmost, Fig. 3(b)]. With no obstructions from the mask, patches of high-speed airflows (i.e., convection zones) are observed that are apparently related to nasal and oral ventilations. With a mask, however, the flow is more widespread on the mask, with elevated speeds near the mask folds [rightmost, Figs. 3(a) vs 3(b)]. Due to the mask resistance, inspiratory airflows are slowed down in the otherwise convective respiration zones. At the same time, as the same amount of air will be inhaled, they approach the oronasal openings through other regions of the mask. With an overall lower speed of carrier airflows, the inhalability of entrained particles can be altered, especially into the nostrils, which orientate downward 30°–45° relative to the gravitational orientation.

Wearing a mask had an insignificant effect on the oronasal flow partition, which was found to be 61:39 (i.e., mouth:nose) with a mask and 60:40 without a mask. The flow Reynolds number (*Re* = *ρUL*/*μ*, with *U* being the local velocity and *L* = 9 mm being the characteristic length at the outlet) was estimated to be 327 in the nose, 520 in the mouth, 965 in the pharynx, 3040 in the glottis, and 2078 at the outlet (trachea).

#### 2. Particle dynamics

**FIG. 4.**
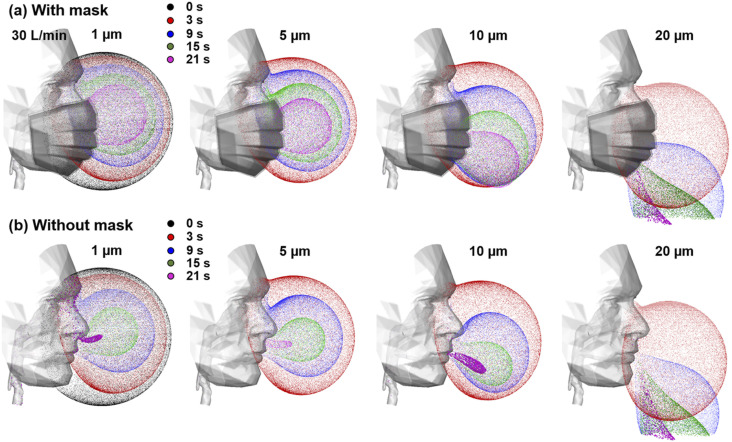
Instantaneous snapshots of particle positions in M0 at different times after their release during (a) the first cycle and (b) the second cycle.

Particle dynamics at an inhalation flow rate of 30 l/min with and without wearing a mask are shown in Fig. 4. First, particles move slower when wearing a mask due to the mask resistance. As a result, particles advance a shorter distance than without wearing a mask during the same period of time [Figs. 4(a) vs 4(b)]. Second, particle behaviors are highly sensitive to the particle size. While 1-*µ*m particles closely follow the inspiratory airflows, large particles of 10 *µ*m and 20 *µ*m exhibit drastically different patterns due to the escalating gravitational effect. As the inhalability of micrometer particles is a tag-war result between the convection and gravitational sedimentation, the presence of a mask, as well as the altered particle motions that are incurred, can perceivably change the particle inhalability, as well as the dosimetry distribution in the downstream airways.

### B. Particle deposition with and without a mask

#### 1. Deposition on the mask

**FIG. 5.**
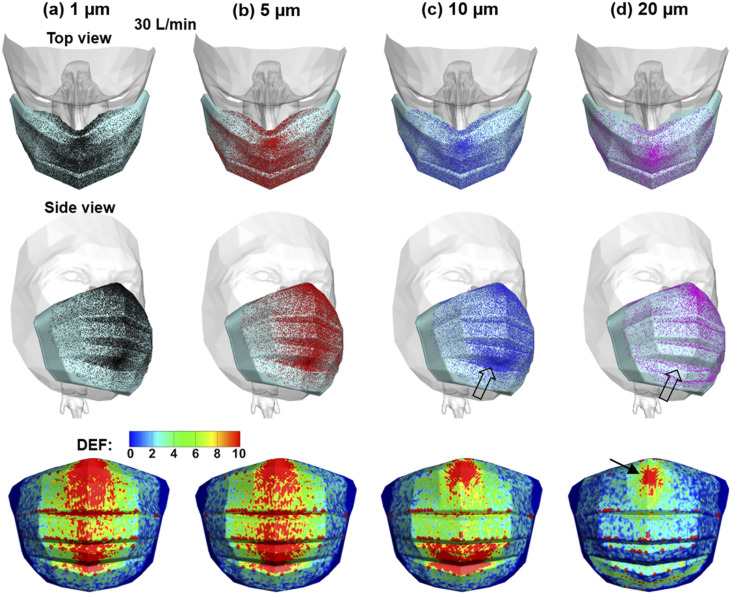
Particle deposition pattern and intensity on the mask at 30 l/min for particles of (a) 1 *µ*m, (b) 5 *µ*m, (c) 10 *µ*m, and (d) 20 *µ*m, with a top view, a side view, and a visualization of particle localizations in terms of the DEF (deposition enhancement factor).

Figure 5 shows the particle deposition on the mask at 30 l/min for various particle sizes. Overall, similar deposition patterns are noted among the particle sizes considered (1 *µ*m, 5 *µ*m, 10 *µ*m, and 20 *µ*m), with subtle variations becoming progressively noticeable with increasing particle size. For instance, few 20-*µ*m particles come in contact with the mask than smaller particles. This decrease is most obvious in the lower folds [hollow arrows, Figs. 5(c) and 5(d)] of the mask. Considering that particle overlapping may prevent an accurate assessment of the dosimetry, the deposition enhancement factor (DEF) was plotted to visualize the deposition intensity as the ratio of the local deposition rate to the average deposition rate. A range of 0–10 was adopted to identify the zones with deposition one order of magnitude higher than the mean dosimetry. As shown in the lower panels of Figs. 5(a)–5(d), elevated deposition occurs along the vertical middle line of the mask, as well as within the mask folding. As the particle size increases, the deposition hot zones decrease in the middle of the mask. For 20-*µ*m particles, elevated dosimetry that is one order of magnitude higher than the mean can only be found at the top of the mask [arrow, lower panel, Fig. 5(d)].

#### 2. Face, upper airway, and lungs

**FIG. 6.**
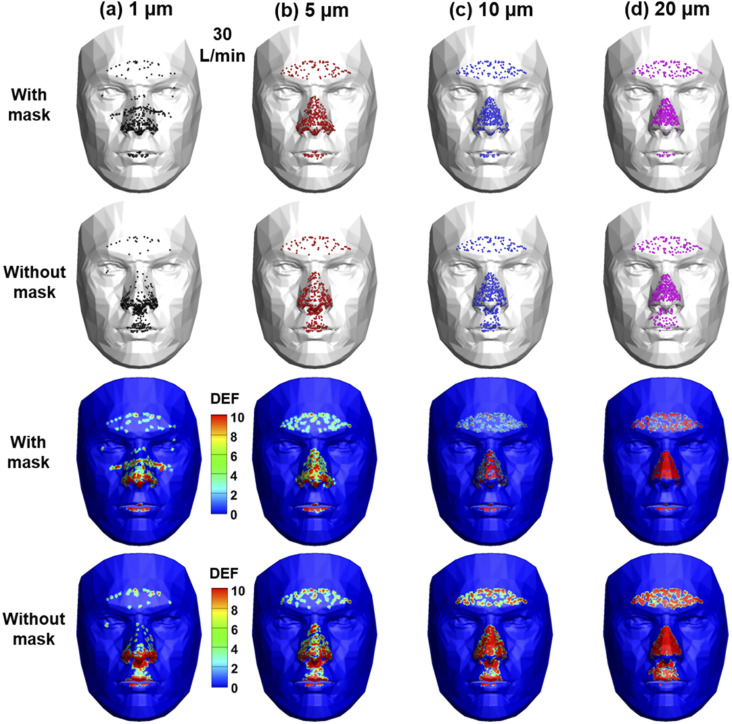
Comparison of the particle deposition pattern and intensity on the face (a) with a mask and without a mask at an inhalation flow rate of 30 l/min for particles of 1 *µ*m, 5 *µ*m, 10 *µ*m, and 20 *µ*m. The deposition intensities were visualized using the DEF (deposition enhancement factor).

A comparison of particle deposition on the face with and without a mask is shown in Fig. 6 for different particle sizes. Smaller particles give rise to more dispersed deposition on the face, regardless of wearing a mask or not. For 1-*µ*m particles, deposition is spotted around the eyes, which are venerable sites of bacterial or viral infections. Interestingly, wearing a mask leads to a higher deposition underneath the eyes [top panel, Fig. 6(a)] than without a mask. As the particle size increases from 1 *µ*m to 20 *µ*m, more particles deposit on the nose and the forehead. In comparison to the deposition with a mask, one major difference is the enhanced deposition in the philtrum, the region below the nose and above the upper lip (second row). The deposition intensities were also compared with and without a mask in terms of the DEF in the third and fourth rows, as shown in Figs. 6(a)–6(d), respectively. Again, the dispersed deposition for 1-*µ*m particles, the increasingly concentrated deposition on the nose and forehead with particle size, and the higher deposition on the philtrum are more vividly displayed with the clear contrast of the DEF colors.

**FIG. 7.**
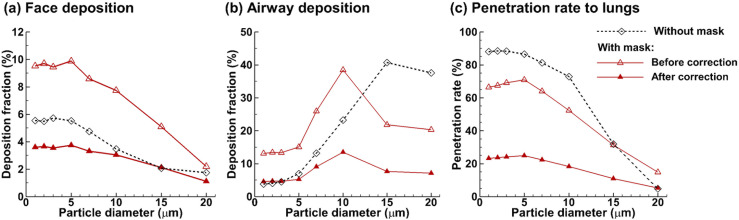
Comparison of the fate of inhaled aerosols at 30 l/min with (red lines) and without (black line) wearing a mask in terms of (a) face deposition, (b) airway deposition, and (c) penetration rate into the lungs. When wearing a mask, two scenarios were considered, with the filtration efficiency being 0% in the first scenario (i.e., before correction, hollow delta, representing the worst limit) and 65% in the second scenario (i.e., after correction, solid delta, representative of a typical three-layer surgical mask). A pass rate of 35% was applied for all particles that came in contact with the outer layer of the mask. For instance, the number of particles depositing on the face was counted as that of particles landing on the uncovered face plus the 35% of particles landing on the face covered by the mask.

Figure 7 compares the dosimetry of ambient aerosols at 30 l/min with and without a mask in terms of face deposition, airway deposition, and penetration rate into the lungs. It is noted that the deposition fraction (DF) with a mask was presented in two formats: (1) “before correction” that assumed zero mask filtration (red hollow delta) and (2) “after correction” that assumed 65% mask filtration (red filled delta). Regarding the face deposition with a mask, the DF after correction was calculated by including 35% of the particles that came in touch with the mask’s outer layer and 100% particles that landed on the uncovered face, as delineated in Fig. 1(b). Much to our surprise, by assuming zero filtration efficiency, wearing a mask leads to significantly higher deposition on the face for all particles considered (1 *µ*m–20 *µ*m) and in the airway for particles of 1 *µ*m–10 *µ*m [Fig. 7(a)]. With a 65% filtration efficiency, which is typical for a three-layer surgical mask, the corrected face deposition falls below the unmasked DFs for all particles, despite a close match for 15-*µ*m particles.

Considering the upper airway [Fig. 7(b)], wearing a zero-filtration mask (red hollow delta) led to a higher deposition of 1 *µ*m–10 *µ*m particles but a lower deposition of 15 *µ*m–20 *µ*m particles than without a mask (blue dotted circle). The corrected airway DF (red filled delta) fell below the unmasked one (blue dotted circle) for particles larger than 3 *µ*m but is still comparable for small particles of size 1 *µ*m–3 *µ*m. Figure 7(c) quantifies the penetration rate of particles into the lungs. In this case, wearing a mask indeed reduces the chance of ambient particles getting into the lungs for all particles considered except for 20 *µ*m ones, which fortunately have very low inhalability due to their weight.

#### 3. Regional deposition in the nose, mouth, pharynx, and larynx

**FIG. 8.**
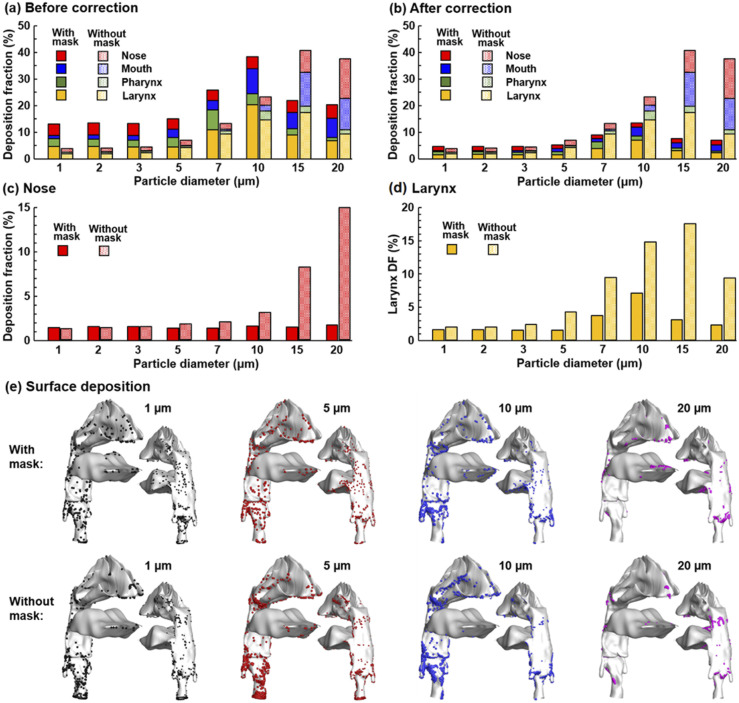
Regional deposition fractions (DFs) at 30 l/min in different sections of the upper airway (i.e., the nose, mouth, pharynx, and larynx): (a) DFs without a mask vs DFs with a mask before correction (with 0% mask filtration efficiency), (b) DFs without a mask vs DFs with a mask after correction (with 65% mask filtration efficiency), (c) the nose DF without a mask vs with a mask after correction, (d) the larynx DF without a mask vs with a mask after correction, and (e) surface deposition in the upper airway for particles of sizes 1 *µ*m, 5 *µ*m, 10 *µ*m, and 20 *µ*m.

Considering that epitheliums in different sections of the respiratory tract have varying susceptibility to inhaled agents, the dosimetry in the upper airway [Fig. 7(b)] was further separated into four regions, nose, mouth, pharynx, and larynx, as shown in Fig. 8(a) (before correction) and Fig. 8(b) (after correction). It is clear that wearing a mask not only changes the overall DF in the upper airway but also the DF distribution among the four regions. Figure 8(c) shows the comparison of the nose deposition with (after correction) and without a mask. In contrast to the high sensitivity of the nasal DF to particle size without a mask, the nasal DF was found to be relatively independent of the particle size when wearing a mask. On the other hand, the laryngeal DF shows high sensitivities, regardless of the presence of a mask. These particle-size-dependent differences highlight the need for future testing of mask filtration efficiency of monodisperse aerosols. Future estimation of infectious respiratory disease transmission and presentation should also consider these size-related discrepancies for more reliable predictions. The surface deposition in the upper airway is shown in Fig. 8(e) for four particle sizes (1 *µ*m, 5 *µ*m, 10 *µ*m, and 20 *µ*m) with and without a mask. Heterogeneous particle distributions are found in all cases hereof, with high levels of particle accumulations in certain areas while no or few particles in other areas. Note the highly different deposition patterns among the four different sized aerosols with a mask shown in the upper panel of Fig. 8(e). Even though the cumulative deposition in the nose may be insensitive to particle size, the local deposition can still be highly sensitive, corroborating the need to study monodisperse aerosols.

### C. Effect of the inhalation flow rate and mask resistance

#### 1. Inhalation rate effects

**FIG. 9.**
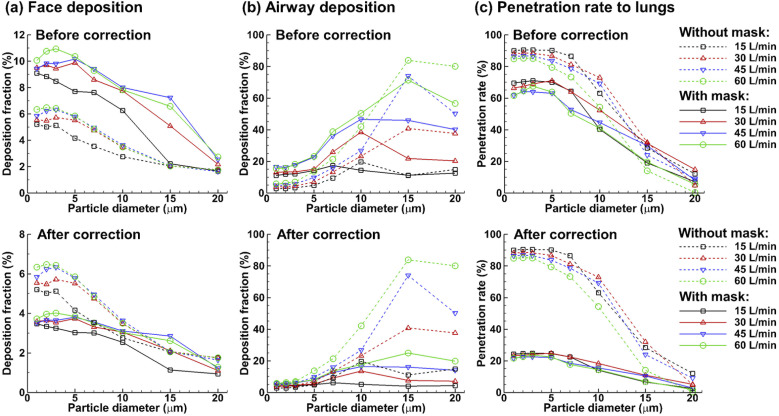
Effects of the flow rate on the fate of inhaled aerosols with (solid lines) and without (dashed lines) wearing a mask in terms of (a) face deposition, (b) airway deposition, and (c) penetration rate into the lungs. The upper panels show the scenario with 0% mask filtration (i.e., before correction), and the lower panel shows the modified rates with a mask filtration efficiency of 65% (i.e., after correction).

**FIG. 10.**
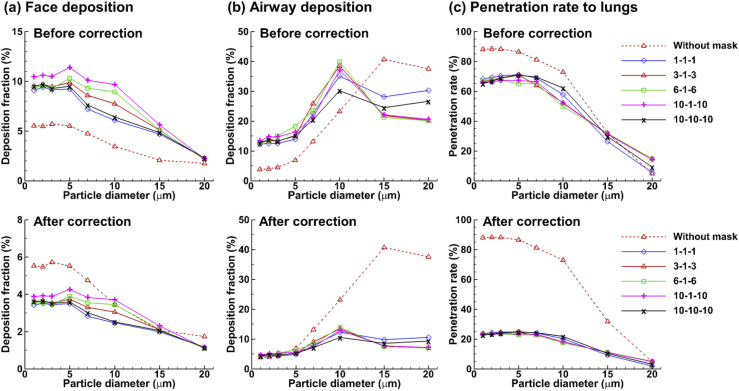
Effects of the mask resistance matrix on the fate of inhaled aerosols at 30 l in comparison to the scenario without a mask: (a) face deposition, (b) airway deposition, and (c) penetration rate into the lungs. The upper panels show the scenario with 0% mask filtration (i.e., before correction), and the lower panel shows that with 65% mask filtration (i.e., after correction).

In the above-mentioned sections, we have examined in detail the dosimetry of ambient aerosols with and without a mask for one flow rate (30 l/min) and one mask (with a resistance matrix of 3-1-3). The impacts from different flow rates and mask resistance matrices were also investigated as presented in Figs. 9 and 10, respectively. Figure 9(a) provides further support that the presence of a zero-filtration mask could lead to higher deposition on the face for micrometer particles. Moreover, this deposition enhancement increases with the increasing inhalation flow rates [upper panel, Fig. 9(a)]. As shown in Fig. 8(a), after considering the 65% mask filtration efficiency, the corrected face dosimetry with a mask falls below that without a mask for all particles, except 15 *µ*m and 20 *µ*m particles [lower panel, Fig. 9(a)]. Similar trends are also observed in Fig. 9(b) for airway deposition, where the uncorrected DFs with a mask are higher than those without a mask for particles smaller than 10 *µ*m and lower for particles of size 15 *µ*m–20 *µ*m. As the inhalation flow rate increases, the deposition in the airway quickly increases for both scenarios, with and without a mask. This increase is especially pronounced for large particles (10 *µ*m–20 *µ*m), whose inhalability is strongly affected by gravity and is increased by intensifying convective effects. Considering Fig. 9(c), increasing the flow rates decreases the penetration rate into the lungs possibly due to the higher filtration efficiency of the upper airway and the associated particle depletion. For all flow rates considered, wearing a mask reduces the lung dosimetry (trachea and below), regardless of the mask filtration efficiency. Wearing a 65%-filtration mask reduces the lung deposition by 2.5–3.5 folds for particles of size 1 *µ*m–10 *µ*m [lower panel, Fig. 9(c)].

#### 2. Mask resistance matrix effects

The effects of the mask resistance matrix on particle dosimetry with and without a mask are shown in Fig. 10 at an inhalation flow rate of 30 l/min. Masks with four additional resistance matrices were considered, i.e., 1-1-1 (homogeneous), 6-1-6, 10-1-10, and 10-10-10 (homogeneous with ten times resistance). Note that 1-1-1 represents a resistance matrix of 8.864 × 10^9^ 1/m^2^ in all three directions, while 10-1-10 represents 8.864 × 10^10^ in lateral directions and 8.864 × 10^9^ in the normal direction. Increasing the lateral resistances (*x* and *z* directions) consistently increases the face deposition, with the highest face DF predicted for the 10-1-10 matrix [Fig. 10(a)]. Similar face DFs were predicted between the two homogeneous masks with a resistance difference of one order magnitude (i.e., 1-1-1 and 10-10-10), as shown in Fig. 10(a). However, consistent lower deposition in the upper airway was predicted with the 10-10-10 matrix than the 1-1-1 matrix [upper panel, Fig. 10(b)] presumably because of the lower particle speeds and the associated lower inhalability of ambient particles after the mask of higher resistance. For all masks considered, significantly lower deposition in the upper airway was predicted by wearing a mask with 65% filtration efficiency for all particles larger than 5 *µ*m [lower panel, Fig. 10(b)]. Insignificant influences from the variation of the resistance matrix were found on the particle penetration rate into the lungs for all micrometer particles considered [Fig. 10(c)].

#### 3. Variation of face deposition

**FIG. 11.**
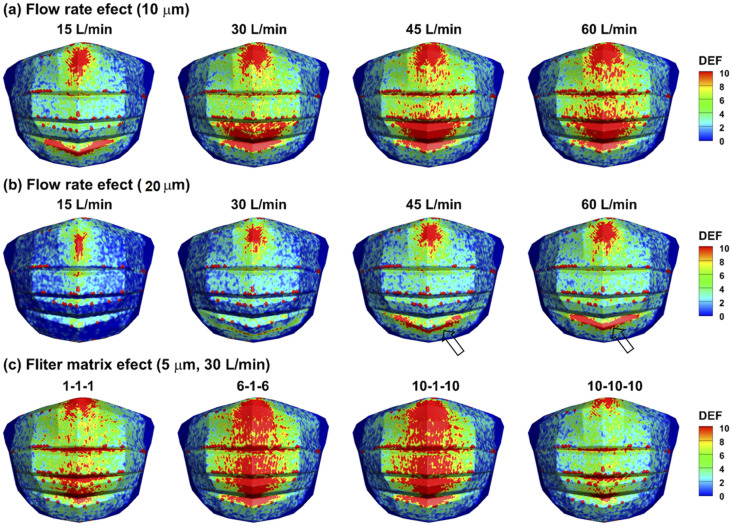
Deposition variation on the mask under different inhalation flow rates for (a) 10-*µ*m particles, (b) 20-*µ*m particles, and (c) with different mask resistances (i.e., filter matrix in three directions: 1-1-1, 6-1-6, 10-1-10, and 10-10-10) for 5-*µ*m particles at 30 l/min.

Figure 11 shows the deposition variation on the mask under the influences of different flow rates and mask resistances. For 10-*µ*m particles [Fig. 11(a)], the deposition hot zones were spotted at the top and bottom of the mask for an inhalation rate of 15 l/min. These two deposition hot zones constantly grew in size with an increasing flow rate from 15 l/min to 60 l/min [Fig. 11(a)]. Deposition in the mask pleats also intensified with increasing flow rates. For 20-*µ*m particles [Fig. 11(a)], similar trends were observed in the deposition hot zones with consistently smaller areas at the corresponding flow rates. Due to a larger gravity effect, a V-shaped deposition hot zone formed in the lower ridge of the third mask folding at 45 l/min and 60 l/min [hollow arrows, Fig. 11(b)]. The effects of mask resistance homogeneity (or deviation from it) on the mask deposition (or the respirable particles passing through the mask) are demonstrated in Fig. 11(c). Compared to the relatively even distribution of particle depositions in the two homogeneous masks (1-1-1 and 10-10-10), intensified deposition occurred along the vertical middle line in the two masks with heterogeneous resistances [Fig. 11(c)].

#### 4. Variation of deposition in the nose, mouth, pharynx, and larynx

**FIG. 12.**
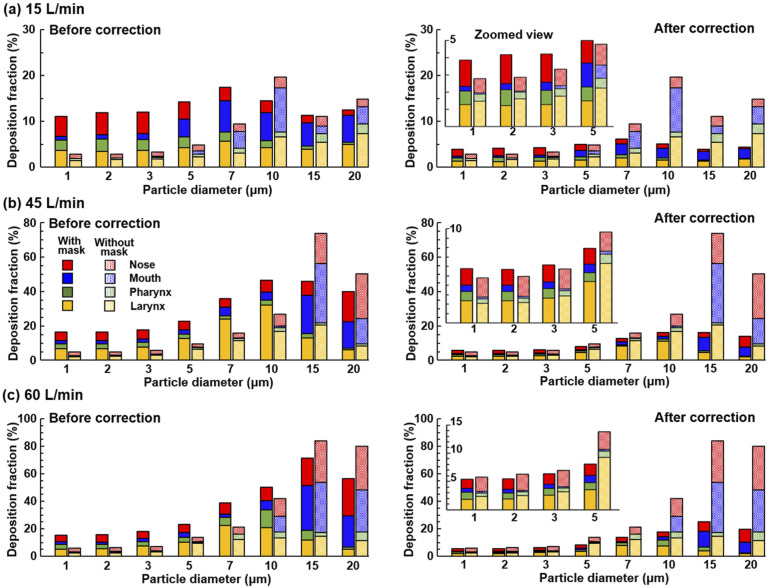
Deposition distribution in different sections of the upper airway (the nose, mouth, pharynx, and larynx) under varying breathing conditions: (a) 15 l/min, (b) 45 l/min, and (c) 60 l/min. The left panels compare the DFs without a mask vs DFs with a mask before correction (with 0% mask filtration efficiency), while the right panels compare the DFs without a mask vs DFs with a mask after correction (with 65% mask filtration efficiency). Zoomed inserts for particles of 1 *µ*m–5 *µ*m are shown in the three right panels.

The effects of the inhalation flow rate on regional DFs in the upper airway are presented in Figs. 12(a)–12(c) for 15 l/min, 45 l/min, and 60 l/min, respectively. Including the case of 30 l/min shown in Fig. 8, it is observed that both the total and regional DFs in the upper airway are sensitive to the inhalation flow rates, which affect the particle inhalability into the airway, as well as the particle transport within the airway, by determining the convective effects vs gravitational sedimentation. Even though the presence of an all-pass mask (i.e., zero filtration efficiency) increases the airway deposition for all flow rates considered, this increase is more significant at lower flow rates for small micrometer particles (right panels, Fig. 12). As a result, noticeably higher DFs were predicted for 1 *µ*m–3 *µ*m particles at 15 l/min by wearing a mask of 65% filtration efficiency than without a mask [the insert panel in Fig. 12(a)]. Even though this abnormality becomes less severe at higher flow rates, the airway DFs are still equivalent in magnitude at 60 l/min with a 65%-filtration mask vs no mask [the insert panel in Fig. 12(c)].

**FIG. 13.**
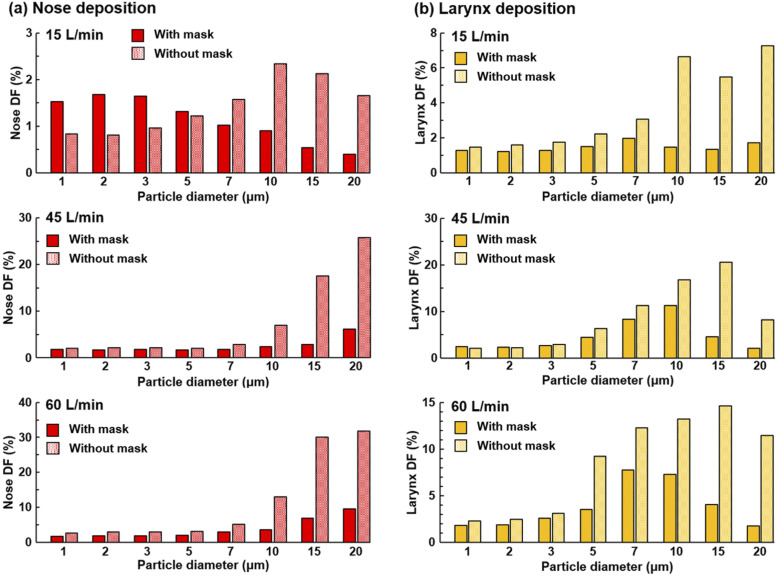
Comparison of the regional airway dosimetry without vs with a mask (after correction) in the (a) nose and (b) larynx at different inhalation flow rates (15 l/min, 45 l/min, 60 l/min).

The dosimetry of ambient aerosols in the nose or larynx can be very different between scenarios with and without a mask at different flow rates. At 15 l/min, the particle deposition in the nose with a 65%-filtration mask peaked at 2 *µ*m and decreased with increasing particle size, whereas the deposition with no mask peaked at 10 *µ*m [top panel, Fig. 13(a)]. As a result, more particles of 1 *µ*m–3 *µ*m deposited in the nose when wearing a mask than when not wearing one. From 30 l/min to 60 l/min, an increasing flow rate persistently enhances the nasal deposition without a mask, while the nasal deposition with a mask shows a much lower sensitivity to the flow rate variation [Figs. 8(c) and 12(a)]. Reminding about the relatively constant nasal DF for different particle sizes at 30 l/min [Fig. 8(a)], the nasal DF increases slightly with particle size at 45 l/min and at a faster pace at 60 l/min; however, both are substantially slower than that without a mask [Fig. 13(a)]. Considering the larynx deposition, the DF peaked at 7 *µ*m–10 *µ*m with a mask and at 15 *µ*m without a mask for all flow rates considered (except for 15 l/min). With a 65%-filtration mask, fewer particles deposited in the larynx for all particle sizes (1 *µ*m–20 *µ*m) and flow rates (15 l/min–60 l/min) considered in this study.

**FIG. 14.**
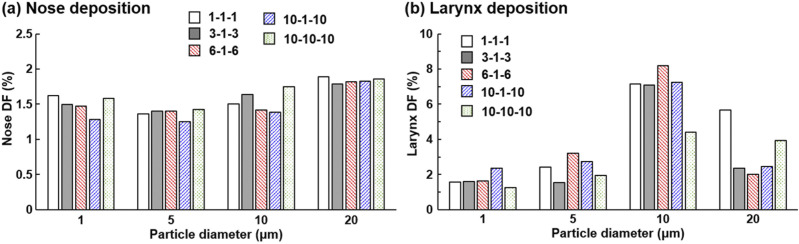
Comparison of the regional airway dosimetry among different mask resistance matrices in the (a) nose and (b) larynx for particles of 1 *µ*m, 5 *µ*m, 10 *µ*m, and 20 *µ*m.

The effect of mask resistance (in terms of homogeneity and magnitude) on particle deposition in the nose and larynx is shown in Figs. 14(a) and 14(b), respectively. The inhalation flow rate was 30 l/min, and four particle sizes were considered (1 *µ*m, 5 *µ*m, 10 *µ*m, and 20 *µ*m). Overall, the mask resistance exerted an insignificant impact on the nasal DF. Minimal DF was observed for the mask with the most heterogeneous resistance (i.e., 10-1-10, blue bar), while similar nasal DFs were predicted for the two homogeneous masks (1-1-1 and 10-10-10) despite a ten times difference in the resistance magnitude. More erratic patterns were found in the larynx deposition with varying mask resistances, with no regular trend detected in light of the mask resistance variation. This lack of regular trend may be partially attributed to the flow instabilities in the pharynx and larynx, where fluctuations in airflow and particle deposition can occur.

## IV. DISCUSSION AND SUMMARY

Since the COVID pandemic from early 2020, the fluids community has been actively involved in elucidating transmission routes of SARS-CoV-2 viruses and devising ways to curb the transmission.^40,41^ Both optical visualization and numerical modeling have been undertaken to understand the respiratory flows and droplets from coughs and sneezes and the effectiveness of face-covering to curtail these droplets.^1,18–20,42–45^ In this study, we aimed at understanding the effect of mask-wearing on inspiratory airflows and their effects on the inhalability and deposition of ambient particles in the upper respiratory airways. A computational mask–face-airway model was developed that consisted of a three-layer surgical mask fitted on the face of an image-based head airway geometry. Factors that influence the inhalability into the nose/mouth and retention in the upper airway and lungs of ambient aerosols were examined, which include (a) with and without a mask, (b) mask filtration efficiency (0% vs 65%), (c) particle size (1 *µ*m–20 *µ*m), (d) inhalation flow rate (15 l/min–60 l/min), and (e) mask resistance (five matrices). We were interested in the dosimetry difference with and without a mask in different regions of the body (the face, upper airway, and lungs) and among the four sections of the upper airway (the nose, mouth, pharynx, and larynx). Mechanisms underlying these differences were explored, and their implications are discussed below.

Wearing a mask was found to notably change the airflow field and particle motions near the face. Due to the mask resistance, the speeds of both airflow and particles decreased in the otherwise respiration zones when no mask was worn; as a compensation, airflow and particles redistribute to regions other than these respiration zones of the mask because the same volume of air will be inhaled with or without a mask (Fig. 3). The overall slowed-down airflow near the face favors the inhalability of particles into the nose, as well as their subsequent deposition in the upper airway. It is also found that the airflow speeds are higher near the folds or pleats of the mask, indicating the potential impacts of mask shape and morphological details on its protective efficacy.

The results of this study show that wearing a zero-filtration mask can lead to a higher deposition rate of particles smaller than 10 *µ*m (i.e., PM10) in the upper airway for all flow rates (15 l/min–60 l/min) and mask resistance matrices considered. This seemingly counterintuitive observation may be attributed to the altered pressure and airflow fields caused by the mask, which further changes the inhalability of the particles and subsequent deposition in the upper airways. The overall lower speeds of the respirable particles after wearing a mask, as well as an increased area of respiration, can increase the chance of respirable particles to land on the face or being inhaled into the mouth and nose. This unexpected finding raises an alarm that wearing masks with very low filtration efficiencies may lead to a higher chance of deposition of ambient aerosols and thus can do more harm than protection. In this study, we assumed a 65% filtration efficiency of the mask, which is typical for a three-layer surgical mask, for all particle sizes. Luckily, the adjusted dosimetry of ambient aerosols is lower with a mask than without one for all particle sizes considered (1 *µ*m–20 *µ*m) in the face, upper airway, and lungs. Considering that the nasal epithelium is one of three sites in the human body binding with the SARS-CoV-2 virus,^46,47^ wearing a 65%-filtration mask can reduce the nasal deposition (viral load) by half for 3 *µ*m–10 *µ*m aerosols and by four to five times for 15-*µ*m aerosols (Fig. 13).

The finding that particle dosimetry can be substantially different with and without a mask calls for cautions in health risk assessment with face coverings. The practice of estimating airway doses with a mask by simply scaling the doses without a mask can introduce significant errors. Furthermore, current mask filtration efficiency (FE) testing, for instance, using TSI 8130, only provides an integrated FE value for polydisperse aerosols and does not differentiate FEs among particle sizes. It is well expected that the mask FE varies significantly with particle sizes. Even though this study adopted an identical FE (65%) for all particles considered, further studies with a mask are warranted to include the mask FEs that are specific to different particle sizes. Likewise, complementary experimental studies are needed to measure the particle-size-dependent FEs for different types of masks.

The nose has a unique role in this COVID-19 pandemic for several reasons. It is the first physical barrier of our body to keep ambient aerosols from getting into the respiratory tract; unlike the mouth, the downward nostrils can effectively prevent large particles from being inhaled due to their large inertia. The nasal mucus and immune cells constitute the second line of defense against invading viruses.^48^ However, the nasal goblet secretory cells are also one of the three confirmed binding sites for COVID-19 viruses, where two necessary enzymes for cell invasion, ACE2 (angiotensin-converting enzyme 2) and TMPRSS2 (type II transmembrane serine protease), coexist.^47^ This explains the usage of nasal swabs in COVID testing.^46^ The other two sites with these two enzymes coexisting are the surface epithelial cells of the alveoli and the ileal absorptive cells in the small intestine.^47^ In this study, we found that the protective efficacy of a mask for the nasal airway decreases at lower inhalation flow rates. Particularly at 15 l/min, the nasal retention of 1 *µ*m–3 *µ*m ambient aerosols is even higher by wearing a 65% filtration mask than without a mask at all. This situation is expected to worsen for flow rates lower than 15 l/min or wearing a mask with lower filtration efficiencies. After saying that, we also wish to emphasize that wearing a 65% filtration mask indeed reduces deposition of ambient aerosols larger than 3 *µ*m on both the face and in all parts of the respiratory tract for all flow rates considered (15 l/min–60 l/min). Moreover, wearing a mask is highly effective in keeping large particles (>10 *µ*m) from getting into the nostrils (i.e., particle inhalability), as illustrated in Figs. 8(c) and 13(a).

Limitations that may compromise the applicability of the results in this study include a perfect seal between the mask and the face, steady breathing, inhalation only, rigid airway walls, and an initial airborne aerosol profile of a spherical shape. It is well known that unlike N95, a disposable three-layer surgical mask does not fit tightly with the face;^49,50^ the fitting can become worse with physical activities or incorrect wearing practices.^51,52^ Air leakages through mask–face spaces can change the airflow and particle dynamics at different levels depending on the location and area of these opening spaces. Using a perfect mask–face seal here cuts the numerous possibilities of such open spaces short and intends to represent the optimal scenario in mask protection from ambient aerosols. However, imperfect mask–face sealing of varying degrees should be investigated to refine the assessment of mask protection efficiencies. Tidal breathing and compliant walls are the other two physiological factors determining respiratory aerodynamics, which further influence the trajectories, inhalability, and deposition of ambient aerosols.^34,53^ Furthermore, interpersonal transmissions of respiratory infectious diseases like COVID-19 are often related to coughs or sneezes from an infected person, which produces a bolus of droplets that vary its shape and size distribution during its transportation through the air.^42,54,55^ In this sense, the spherical profile of monodisperse particles adopted in this study may not adequately represent the interpersonal transmissions. Moreover, the hygroscopic effects and electrostatic charges were excluded, both of which had been demonstrated to change the particle fates and behaviors.^56–60^ However, the computational model herein did take into account the most fundamental properties affecting a mask’s performance, such as a realistic mask model with morphological details (folds) and experimentally determined properties (filtration efficiency and breathing resistance), an anatomically accurate face-airway geometry, and ambient aerosols are representative of COVID-19 virus-laden droplets.^8^ With the assumptions of a perfect mask–face interface, constant inhalation, non-moving walls, and monodisperse particles that greatly reduced numerical complexities, the results of this study provide a first-order approximation of mask performance in real life. Likewise, the computational model developed in this study can serve as a platform where more physiologically realistic factors can be evaluated.

In summary, the effects of wearing a three-layer surgical mask on airflow and aerosol dynamics were examined in a mask–face-airway model in comparison to without a mask. A better understanding of the factors involved in determining the dosimetry of ambient aerosols on the face and in the respiratory tract was obtained. Specific findings are as follows:

1. Wearing a mask significantly slows down inspiratory flows and extends respiration zones, which favors the inhalability of ambient aerosols into noses.
2. High flow speed and elevated particle concentrations are observed in the mask pleats.
3. Wearing a mask significantly reduces particle penetration into the lungs, regardless of the filtration efficiency of the mask. Wearing a 65%-filtration mask can reduce lung deposition by three folds for particles of size 1 *µ*m–10 *µ*m.
4. With a 65% mask filtration efficiency that is typical for a three-layer surgical mask, deposition is reduced by wearing a mask for all particle sizes considered, except 1 *µ*m–3 *µ*m, for which equivalent dosimetry in the upper airway was predicted.
5. Wearing a mask protects the upper airway (particularly the nose and larynx) best from particles larger than 10 *µ*m, while it protects the face and lungs best from particles less than 10 *µ*m (PM10).
6. The mask protection of the nasal airway, whose goblet secretory cells are binding sites for SARS-CoV-2, decreases at lower inhalation flow rates (15 l/min or less).

## Data Availability

The data that support the findings of this study are available from the corresponding author upon reasonable request.
